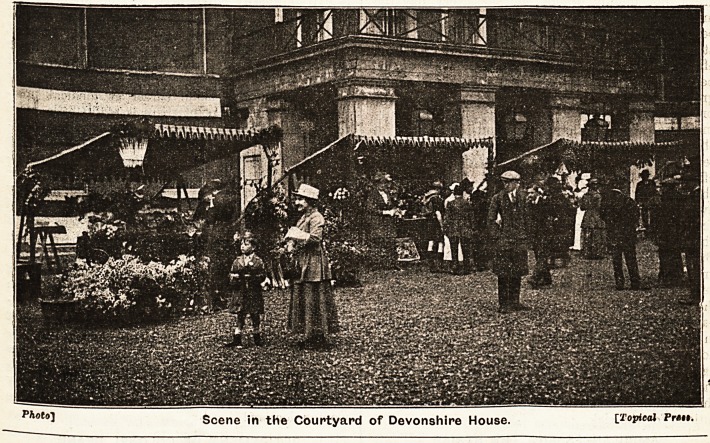# The Red Cross Market

**Published:** 1920-10-23

**Authors:** 


					Ostobbr 23; 1920. THE HOSPITAL. 67
The Red Cross Market.

				

## Figures and Tables

**Figure f1:**